# Supplementary material for: In vivo Hox binding specificity revealed by systematic changes to a single cis regulatory module
Source: Nat Commun. 2019 Aug 9;10:3597. doi: 10.1038/s41467-019-11416-1 (PMC6689074; doi:10.1038/s41467-019-11416-1)
Supplement: Supplementary file 1 — Supplementary Information [file 41467_2019_11416_MOESM1_ESM.pdf]

Supplementary Information

In vivo Hox binding specificity revealed by systematic changes to a single cis regulatory module

Carlos Sánchez-Higueras et al.

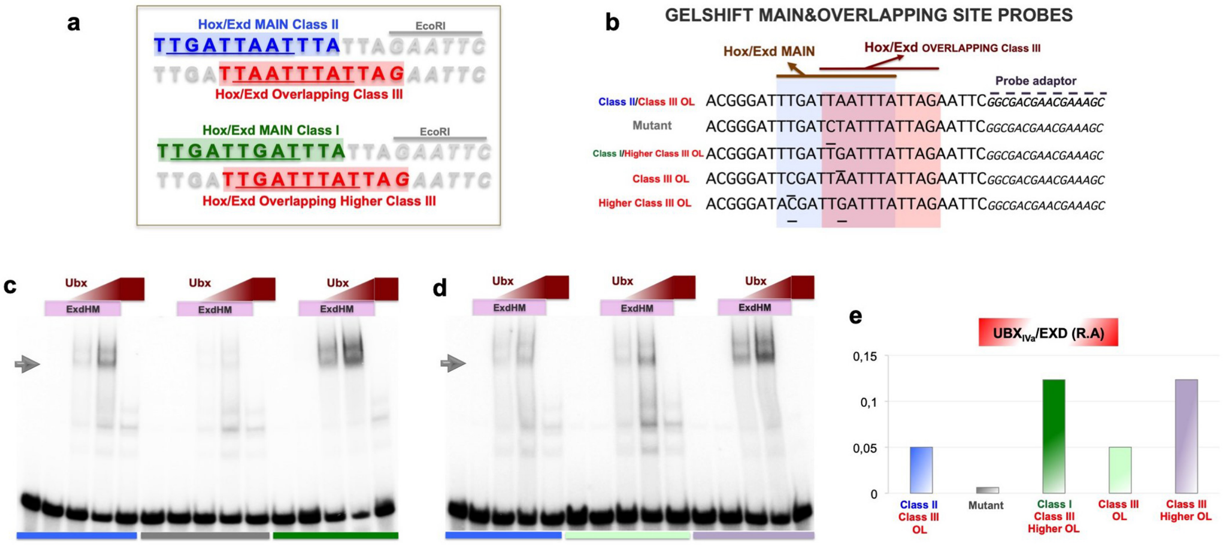

Supplementary Figure 1: Biochemical and *in silico* predicted site affinity to *main* and *overlapping* class 3 sites or to the *overlapping* site

(a) Sequence comparison of the oligo containing the *main* and *overlapping* wild type sites with that carrying the class 1 mutated site (green) that secondarily generates a higher affinity class 3 site. (b) oligo probes used to test in EMSA the affinity to probes containing variants for both sites or only containing the *overlapping* site after mutation of the main site.
(c) Ubx binding to both the *main* class 2 site and *overlapping* class 3 containing oligo (blue lanes); to a mutant for both sites (grey); to a *main* class 1 site that transformed the *overlapping* site into a High class 3 (green). (d) Ubx binding to both the *main* class 2 site and *overlapping* class 3 containing oligo (blue); to a mutant *main* with a class 3 *overlapping* site (light green); or to a mutant *main* with a high affinity class 3 *overlapping* site (light purple). (e) *In silico* predicted affinity of Ubx to sites shown in (c-d).
Comparison of blue lanes with light green labelled lanes in d shows Ubx binding is maintained or even increased in the absence of the *main* site, indicating Ubx binding to the oligos containing two sites is mostly occurring through the *overlapping* class 3 site. This conclusion is reinforced by the strong binding of Ubx observed in the light purple labelled lanes where only the *overlapping* site remains. These EMSA results indicate that the trunk expression observed in *S1+55cl1* embryos is mediated through the *overlapping* site. Source data are provided as a Source Data file.

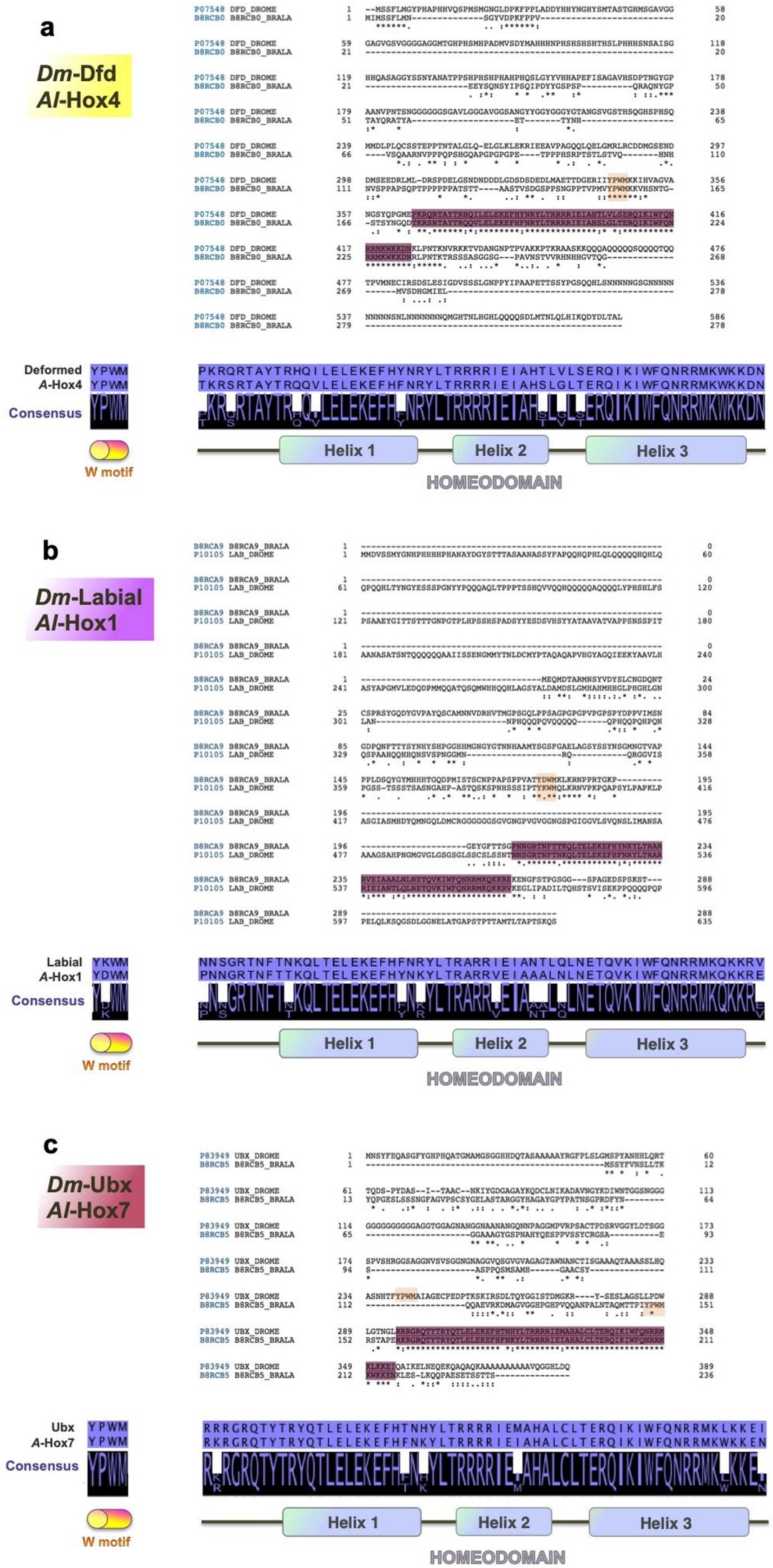
a
Dm-Dfd
Al-Hox4
Deformed YPWM
A-Hox4 YPWM
Consensus
W motif
PKRQRTAYTRHQILELEKEFHYNRYLTRRRRIEIAHTLVLSERQIKIWFQNRRMKWKKDN
TKRSRTAYTRQQVLELEKEFHFNRYLTRRRRIEIAHSLGLTERQIKIWFQNRRMKWKKDN
Helix 1
Helix 2
Helix 3
HOMEODOMAIN
b
Dm-Labial
Al-Hox1
Labial YKWM
A-Hox1 YDWM
Consensus
W motif
NNSGRTNFTNKQLTELEKEFHFNRYLTRARRIEIANTLQLNETQVKIWFQNRRMKQKKRV
PNNGRTNFTTKQLTELEKEFHYNKYLTRARRVEIAAALNLNETQVKIWFQNRRMKQKKRE
Helix 1
Helix 2
Helix 3
HOMEODOMAIN
c
Dm-Ubx
Al-Hox7
Ubx YPWM
A-Hox7 YPWM
Consensus
W motif
RRRGRQTYTRYQTLELEKEFHTNHYLTRRRRIEMAHALCLTERQIKIWFQNRRMKLKKEI
RKRGRQTYTRYQTLELEKEFHFNKYLTRRRRIEIAHALCLTERQIKIWFQNRRMKWKKEN
Helix 1
Helix 2
Helix 3
HOMEODOMAIN

Supplementary Figure 2: Sequence alignment of *D. melanogaster* and *A. lanceolatus* orthologous proteins

Full sequence Uniprot alignments of (a) Dfd and Al-Hox4, (b) Lab and Al-Hox1 and (c) Ubx and Al-Hox7. In all cases most conservation maps to the homeodomain especially on the DNA-contacting third helix and on the cofactor interacting hexapeptide motif. Specific alignments of the hexapeptide motif (also known as W motif) and the homeodomain performed with Jalview are shown under the full sequence alignment

Note that the most similar cofactor interacting motives (labelled in orange) between UbxIVa and Al-Hox7 do not align when using either Uniprot or Clustal w software with default settings. UbxIVa contains 4 described W motifs [35].

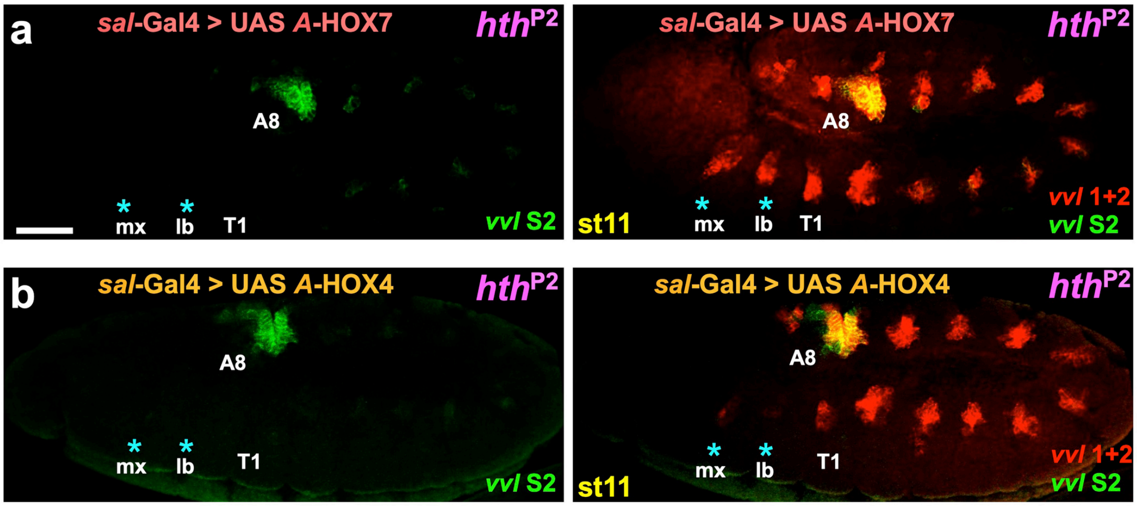

Supplementary Figure 3: *Amphioxus* Hox monomeric function in *Drosophila*

Expression of *S2-GFP* (a-b) or *vvl1+2-mCherry* (red) and *S2-GFP* (a-b, right panels) in *hth*^{P2} mutant embryos that express in the maxilla and labium (asterisks) the *UAS-AHox7* (a) or *UAS-AHox4* (b) with *sal-Gal4.* AHox-7 can rescue *vvl1+2* but not *S2* expression, while AHox-4 cannot rescue any of them. We did not test the rescue capacity of AHox1 in *hth*^{P2} embryos as AHox1 is unable to rescue any of these constructs in a wild type embryo (see Fig. 4l). Scalebar 50μm.

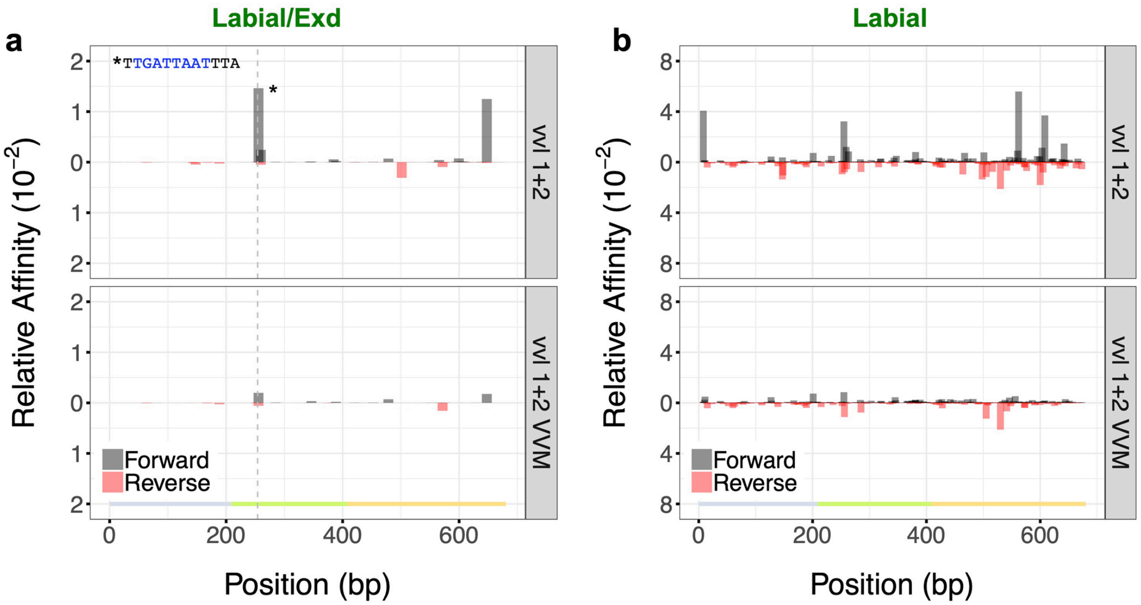

Supplementary Figure 4: Predicted site affinity of Lab to *vvl1+2* and *vvl1+2vvm*

NRLB predicted relative affinity of Lab-Exd (a) or of monomer Lab (b) to *vvl1+2* or to the remaining Hox binding sites in the *vvl1+2vvm*. Asterisk and discontinuous vertical line in (a) label the position of the *main* site.

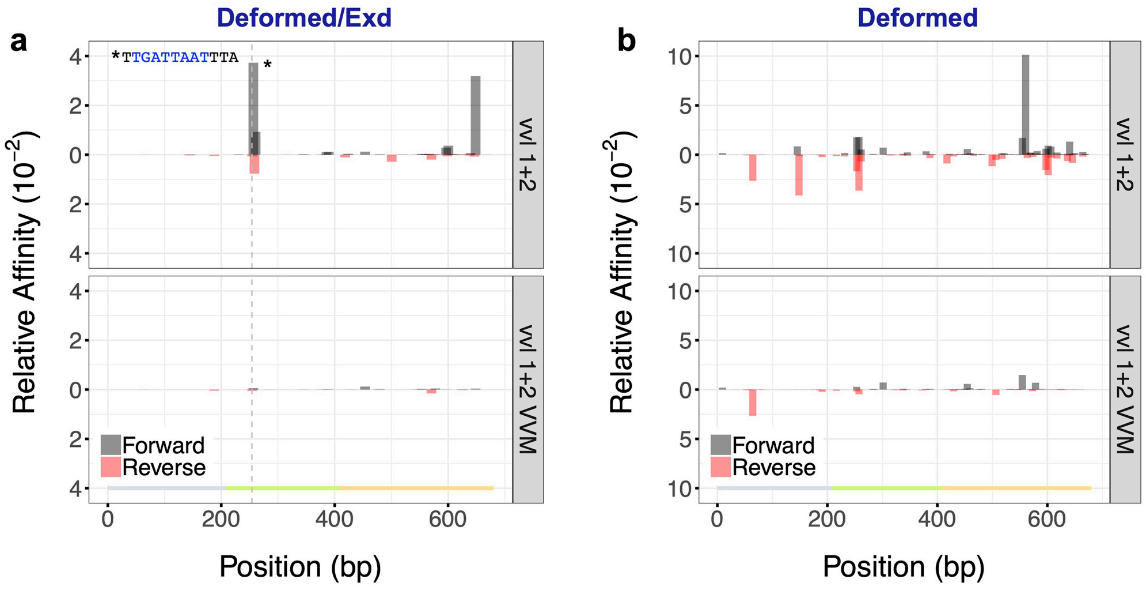

Supplementary Figure 5: Predicted site affinity of Dfd to *vvl1+2* and *vvl1+2vvm*

NRLB predicted relative affinity of Dfd-Exd (a) or of monomer Dfd (b) to *vvl1+2* or to the remaining Hox binding sites in the *vvl1+2vvm*. Asterisk and discontinuous vertical line in (a) label the position of the *main* site.

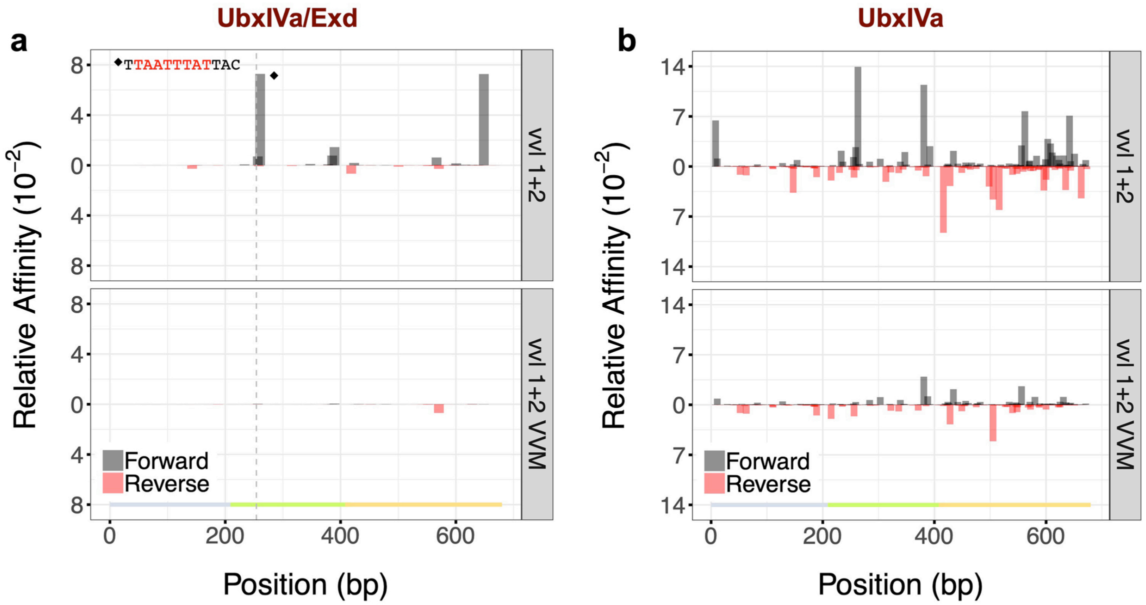

Supplementary Figure 6: Predicted site affinity of Ubx IVa to *vvl1+2* and *vvl1+2vvm*

NRLB predicted relative affinity of UbxIVa-Exd (a) or of monomer UbxIVa (b) to *vvl1+2* or to the remaining Hox binding sites in the *vvl1+2vvm*. Diamond and discontinuous vertical line in (a) label the position of the *overlapping* site.

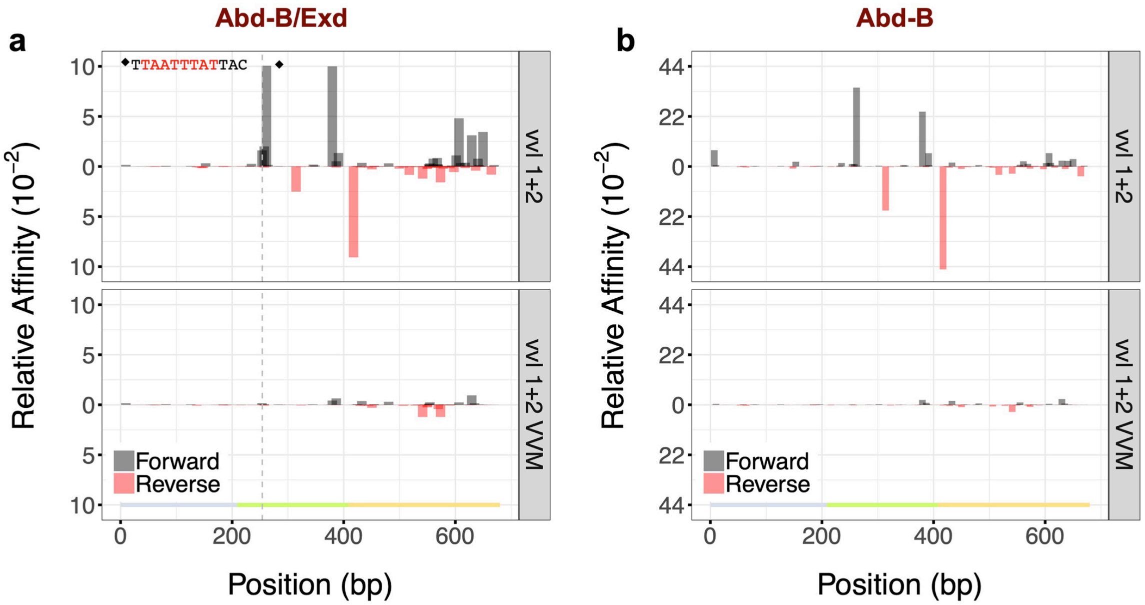

Supplementary Figure 7: Predicted site affinity of Abd-B to *vvl1+2* and *vvl1+2vvm*

NRLB predicted relative affinity of AbdB-Exd (a) or of monomer AbdB (b) to *vvl1+2* or to the remaining Hox binding sites in the *vvl1+2vvm*. Diamond and discontinuous vertical line in (a) label the position of the *overlapping* site.

```
vvl 1+2       GGTTAATGATGGCCACACAGGCGACAACTGGCGACTGAGATCTTGTCCCGCAATTTCCCG   60
              ||||##||||||||||||||||||||||||||||||||||||||||||||||||||||||
vvl 1+2 vvm   GGTTggTGATGGCCACACAGGCGACAACTGGCGACTGAGATCTTGTCCCGCAATTTCCCG   60

vvl 1+2       ATCATTTGCTCAGATACGATACGGATTCTGCGAAGTCACCGGGCAAAGGCATCCCACTGA   120
              ||||||||||||||||||||||||||||||||||||||||||||||||||||||||||||
vvl 1+2 vvm   ATCATTTGCTCAGATACGATACGGATTCTGCGAAGTCACCGGGCAAAGGCATCCCACTGA   120

vvl 1+2       GTTTGTTGGCTTCTTGTCAAACAAATCATTTACTGCGGATACTCCTCGATATTCCTCAAG   180
              |||||||||||||||||||||||||#||###|||||||||||||||||||||||||||||
vvl 1+2 vvm   GTTTGTTGGCTTCTTGTCAAACAAAgCAgggACTGCGGATACTCCTCGATATTCCTCAAG   180

vvl 1+2       ATACTATCCATTTCACTGTGTAGGTGTGAGCTGCAATTTTCCCTGGAAAAATTACGTCCA   240
              |||||||||||||||||||||||||||||||||||||||||||||||||||##|||||||
vvl 1+2 vvm   ATACTATCCATTTCACTGTGTAGGTGTGAGCTGCAATTTTCCCTGGAAAAAggACGTCCA   240

vvl 1+2       GCACGGGATTTGATTAATTTATTACCGCTTGTCGAAGGGAAAGTGATCTTCGGGTTCCTA   300
              |||||||||||||#|||||#||#|||||||||||||||||||||||||||||||||||||
vvl 1+2 vvm   GCACGGGATTTGATcAATTTcTTcCCGCTTGTCGAAGGGAAAGTGATCTTCGGGTTCCTA   300

vvl 1+2       ACGGTTGCGGATCGTAAAAAACATTCGGCAGACACAATTGTTGAATTATCTGCGGGCTGC   360
              |||||||||||#||#||||||||||||||||||||||||||||||##|||||||||||||
vvl 1+2 vvm   ACGGTTGCGGAgCGgAAAAAACATTCGGCAGACACAATTGTTGAAggATCTGCGGGCTGC   360

vvl 1+2       TGTTGTGGGCACTTTTTTATGAGTTATTTATGTGCGACTGTGGCGCCAACAGGATCATAA   420
              |||||||||||||||#|||||#|||||#||||||||||||||||||||||||||||||##
vvl 1+2 vvm   TGTTGTGGGCACTTTgTTATGgGTTATgTATGTGCGACTGTGGCGCCAACAGGATCATgg   420

vvl 1+2       AATATGTAGTTATGGGTAAATCTGTGAAAATAAATGTAAGCGTAATCTGGAAAATATTGA   480
              |||||||||||||||||||||||||||||||||||||||||||##|||||||||||||||
vvl 1+2 vvm   AATATGTAGTTATGGGTAAATCTGTGAAAATAAATGTAAGCGTggTCTGGAAAATATTGA   480

vvl 1+2       GTGGCCTAGATTCACCACATTAGTCATTTCAAAGAAATTAAAGAAAACTAAAGTAGAAGT   540
              |||||||||||||||||||##||||||||||||||||##|||||||||||||||||||||
vvl 1+2 vvm   GTGGCCTAGATTCACCACAggAGTCATTTCAAAGAAAggAAAGAAAACTAAAGTAGAAGT   540

vvl 1+2       ATAAAAATTTTAAATTGTAATGATTTAAATATATCAAATAATGCAAATTTTAAAAAATTA   600
              ||||||||||||||||||##|||###|||||||||||||##||||||||||||||||##|
vvl 1+2 vvm   ATAAAAATTTTAAATTGTggTGAgggAAATATATCAAATggTGCAAATTTTAAAAAAggA   600

vvl 1+2       ATTTGATTACTGCTTTATTAAAATTTTTATTTCATATTAATATGATTTATCGTGCATACG   660
              ||||||##|||||||||##|||||||||||||||||##|||||||###||||||||||||
vvl 1+2 vvm   ATTTGAggACTGCTTTAggAAAATTTTTATTTCATAggAATATGAgggATCGTGCATACG   660

vvl 1+2       GAAATTAAACTGGATTATGG   680
              ||||##||||||||##||||
vvl 1+2 vvm   GAAAggAAACTGGAggATGG   680
```

S1: [1 → 209]
S2: [210 → 418]
S3: [419 → 680]

***vvl* 1+2 genomic location: (Chr3L: 6,764,714-6,765,393)**

Supplementary Figure 8: Aligned *vvl1+2* and *vvl1+2vvm* sequences

The *vvl1+2* sequence aligned to the *vvl1+2vvm* sequence indicating the mutated sites. The *S1* sequence is shown in grey, *S2* in green and *S3* in brown. Genomic location coordinates for the *vvl1+2* enhancer are included.

REPORTER GENE	Intercalary Segment (ic)	Maxillary Segment (mx)	Labial Segment (lb)	Trunk Segments (T2-A7)	Eighth Abdominal Segment (A8)	MAIN&OL Hox/Exd sites	Global Hox Monomer Input per fragment
vvl 1+2	☒	☑☑☑☑	☑☑☑☑	☑☑☑☑	☑☑☑☑	Main cl2<br>OL cl3	high
vvl S1+S2	☒	☑☑☑☑	☑☑☑☑	☑☑☑☑	☑☑☑☑	Main cl2<br>OL cl3	high
vvl S2	☒	☑☑☑	☒	☑☑☑☑	☑☑☑☑	Main cl2<br>OL cl3	high
vvl S1+55	☒	☑☑	☑☑☑	☒	☒	Main cl2<br>OL cl3	weak
vvl S1+55 cl1	☑☑	☑☑☑	☑☑☑☑	☑	☒	Main cl1<br>Higher OL cl3	weak
vvl S1+55 cl3	☒	☑	☑☑☑	☑	☑	Main cl3	weak
vvl S1+55 cl1OF	☑☑☑☑	☑☑☑	☑☑☑	☒	☒	Main cl1OF	weak
vvl S1+55 cl2OF	☒	☑☑☑☑	☑☑☑☑	☒	☒	Main cl2OF	weak
vvl S1+55 cl3OF	☒	☑☑☑	☑☑☑	☑☑	☑☑☑	Main cl3OF	weak
vvl S1+55 mut	☒	☒	☒	☒	☒	none	weak
vvl 1+2 Main&OL mut	☒	☑	☑	☑☑☑	☑☑☑☑	none	high
vvl S2 Main&OL mut	☒	☒	☒	☒	☑☑☑☑	none	high
vvl 1+2 vvm	☒	☒	☒	☒	☒	none	weak
vvl 1+2 vvm-R	☒	☑☑☑	☑☑☑	☑☑☑☑	☑☑☑☑	Main cl2<br>OL cl3	weak
vvl S1	☒	☒	☒	☒	☒	none	weak
vvl S3	☒	☒	☒	☒	☒	none	high

☑☑☑☑ Highest optimal expression ☑☑☑ High expression ☑☑ Medium expression ☑ Low Expression ☒ Occasional not significant expression

Supplementary Table 1: Summary of the segmental expression in different reporter genes

At a glance summary of the expression driven by the different reporters. The levels of expression in each segment are indicated using a subjective five degree scale: occasional not significant expression; low expression; medium expression; high expression; highest optimal expression.
The presence or absence of the *main* and *overlapping* sites and the binding class to which they belong, plus the abundance of monomer sites in each reporter are indicated in the last two columns.

EMSA gel Figure 3i

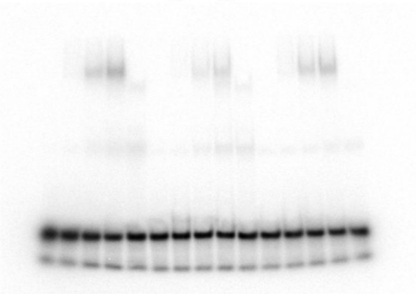

EMSA gel Figure 3j

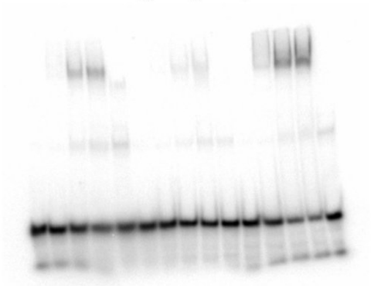

EMSA gel Figure 3l

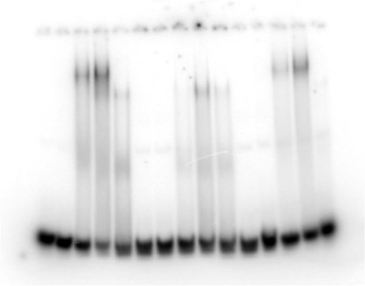

EMSA gel Figure 3m

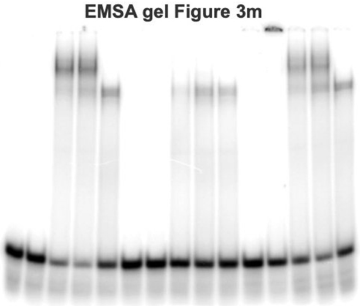

EMSA gel Figure 3o

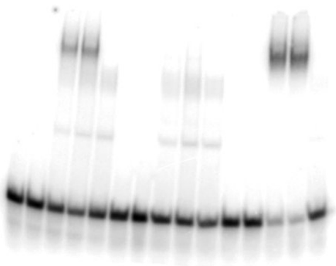

EMSA gel Figure 3p

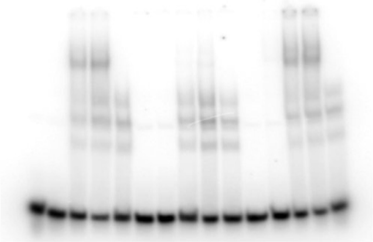

EMSA gel Figure 3r

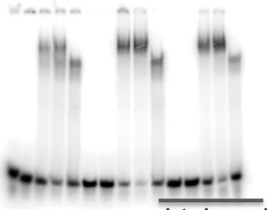

unrelated experiment

EMSA gel Figure 3t

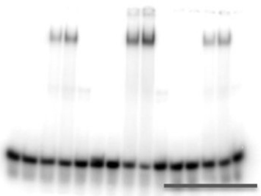

unrelated experiment

EMSA gel Figure 3v

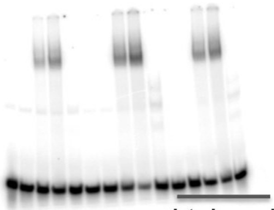

unrelated experiment

EMSA gel Supplementary Figure1c

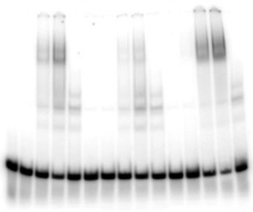

EMSA gel Supplementary Figure1d

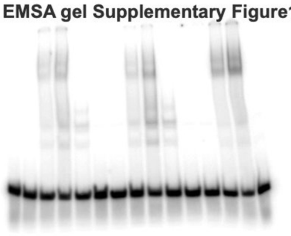

Unprocessed scans of EMSA experiments

Primer List

vvl S1+55

Forward *vvl* S1 EcoRI:
5’-AAAGAATTCGGTTAATGATGGCCACACAGGCGAC-3’

Rvs *vvl* S1+55 EcoRI:
5’-AAAGAATTCTAATAAATTAATCAAATCCCGTGCTGGACGTAA-3’

---

vvl S1+55 CI1

Forward *vvl* S1 EcoRI:
5’-AAAGAATTCGGTTAATGATGGCCACACAGGCGAC-3’

Reverse *vvl* S1+55 CI1 EcoRI:
5'-AAAGAATTCTAATAAATCAATCAAATCCCGTGCTGGACGTAA-3'

---

vvl S1+55 CI3

Forward *vvl* S1 EcoRI:
5’-AAAGAATTCGGTTAATGATGGCCACACAGGCGAC-3’

Reverse *vvl* S1+55 CI3 EcoRI:
5’-AAAGAATTCTAATAAATAAATCAAATCCCGTGCTGGACGTAA-3’

---

vvl S1+55 CI1 OF

Forward *vvl* S1 EcoRI:
5’-AAAGAATTCGGTTAATGATGGCCACACAGGCGAC-3’

Reverse *vvl* S1+55 CI1 OF EcoRI:
5'-AAAGAATTCTAAGTAATCAATCATATCCCGTGCTGGACGTAA-3'

---

vvl S1+55 CI2 OF

Forward *vvl* S1 EcoRI:
5’-AAAGAATTCGGTTAATGATGGCCACACAGGCGAC-3’

Reverse S1+55 CI2 OF:
5'- AAAGAATTCTAAGTCATTAATCATATCCCGTGCTGGACGTAA-3'

---

vvl S1+55 CI3 OF

Forward *vvl* S1 EcoRI:
5’-AAAGAATTCGGTTAATGATGGCCACACAGGCGAC-3’

Reverse *vvl* S1+55 Cl3 OF EcoRI:
5’-AAAGAATTCTAAGTCATAAATCATATCCCGTGCTGGACGTAA-3’

---

vvl S1+55 mutant

Forward *vvl* S1 EcoRI:
5’-AAAGAATTCGGTTAATGATGGCCACACAGGCGAC-3’

Reverse *vvl* S1+55 mutant EcoRI:
5’-AAAGAATTCTAATAAATTGATCAAATCCCGTGCTGGACGTAA-3’

---

vvl S2

Forward *vvl* S2 EcoRI :
5':AAAGAATTCGCTGCAATTTTCCCTGGAAAAATTACGTCCAGCACGGGATTTGATTAATTTATTA-3'

Rvs S2 EcoRI:
5'-AAAGAATTCATGATCCTGTTGGCGCCACAGTCGCACAT-3'

---

vvl S2 MAIN&OL mutant

Forward *vvl* S2 Main&OL mut EcoRI:
5'-AAAGAATTCGCTGCAATTTTCCCTGGAAAAATTACGTCCAGCACGGGATTTGATCAATTTATTA-3'

Rvs S2 EcoRI:
5'-AAAGAATTCATGATCCTGTTGGCGCCACAGTCGCACAT-3'

---

vvl S1+S2

Forward *vvl* S1 EcoRI:
5’-AAAGAATTCGGTTAATGATGGCCACACAGGCGAC-3’

Rvs S2 EcoRI:
5'-AAAGAATTCATGATCCTGTTGGCGCCACAGTCGCACAT-3'

---

vvl 1+2 MAIN&OL mutant*

External Forward *vvl* S1 EcoRI:
5’-AAAGAATTCGGTTAATGATGGCCACACAGGCGAC-3’

External Reverse *vvl* S3 EcoRI:
5’-AAAGAATTCCCATAATCCAGTTTAATTTCCGTATGCACGATAAA-3’

Forward *vvl* S2 Main&OL mutant:
5'- GATCAATTTCTTCCCGCTTGTCGAAGGGAAAGTG-3'

Reverse *vvl* S2 Main&OL mutant:
5'-CCTTCGACAAGCGGGAAGAAATTGATCAAATCCCGTGCTGG-3'

*** PCR mutagenesis in two steps (First Round PCRs and Second Round of Overlapping PCRs).**

---

vvl 1+2 *vvm*-R*

External Forward *vvl* S1 *vvm* EcoRI:
5'-AAAGAATTCGGTTGGTGATGGCCACACAGGCGACAA-3'

External Reverse *vvl* S3 *vvm* EcoRI:
5'-AAAGAATTCCCATCCTCCAGTTTCCTTTCCGTATGCAC-3'

Fwd *vvl* S2 Main&OL restored:
5'-AGCACGGGATTTGATTAATTTATTACCGCTTGTC-3'

Rvs *vvl* S2 Main&OL restored:
5'-GACAAGCGGTAATAAATTAATCAAATCCCGTGCT-3'

*** PCR mutagenesis in two steps (First Round PCRs and Second Round of Overlapping PCRs)**
