# Supplementary material for: In vivo Hox binding specificity revealed by systematic changes to a single cis regulatory module
Source: Nat Commun. 2019 Aug 9;10:3597. doi: 10.1038/s41467-019-11416-1 (PMC6689074; doi:10.1038/s41467-019-11416-1)
Supplement: Supplementary file 3 — Reporting Summary [file 41467_2019_11416_MOESM3_ESM.pdf]

nature research

Corresponding author(s): C. Sanchez-Higueras,R. Mann, J. Hombría

Last updated by author(s): Jun 19, 2019

Reporting Summary

Nature Research wishes to improve the reproducibility of the work that we publish. This form provides structure for consistency and transparency in reporting. For further information on Nature Research policies, see Authors & Referees and the Editorial Policy Checklist.

Statistics

For all statistical analyses, confirm that the following items are present in the figure legend, table legend, main text, or Methods section.

n/a	Confirmed	
☒	☐	The exact sample size (*n*) for each experimental group/condition, given as a discrete number and unit of measurement
☒	☐	A statement on whether measurements were taken from distinct samples or whether the same sample was measured repeatedly
☒	☐	The statistical test(s) used AND whether they are one- or two-sided<br>*Only common tests should be described solely by name; describe more complex techniques in the Methods section.*
☒	☐	A description of all covariates tested
☒	☐	A description of any assumptions or corrections, such as tests of normality and adjustment for multiple comparisons
☒	☐	A full description of the statistical parameters including central tendency (e.g. means) or other basic estimates (e.g. regression coefficient) AND variation (e.g. standard deviation) or associated estimates of uncertainty (e.g. confidence intervals)
☒	☐	For null hypothesis testing, the test statistic (e.g. *F*, *t*, *r*) with confidence intervals, effect sizes, degrees of freedom and *P* value noted<br>*Give P values as exact values whenever suitable.*
☒	☐	For Bayesian analysis, information on the choice of priors and Markov chain Monte Carlo settings
☒	☐	For hierarchical and complex designs, identification of the appropriate level for tests and full reporting of outcomes
☒	☐	Estimates of effect sizes (e.g. Cohen's *d*, Pearson's *r*), indicating how they were calculated

Our web collection on statistics for biologists contains articles on many of the points above.

Software and code

Policy information about availability of computer code

Data collection	No new software was used. All software used has been previously published and the reference is provided.
Data analysis	*Provide a description of all commercial, open source and custom code used to analyse the data in this study, specifying the version used OR state that no software was used.*

For manuscripts utilizing custom algorithms or software that are central to the research but not yet described in published literature, software must be made available to editors/reviewers. We strongly encourage code deposition in a community repository (e.g. GitHub). See the Nature Research guidelines for submitting code & software for further information.

Data

Policy information about availability of data

All manuscripts must include a data availability statement. This statement should provide the following information, where applicable:

- Accession codes, unique identifiers, or web links for publicly available datasets
- A list of figures that have associated raw data
- A description of any restrictions on data availability

All relevant data are available from the authors

Field-specific reporting

Please select the one below that is the best fit for your research. If you are not sure, read the appropriate sections before making your selection.

☒ Life sciences ☐ Behavioural & social sciences ☐ Ecological, evolutionary & environmental sciences

Life sciences study design

All studies must disclose on these points even when the disclosure is negative.

Sample size	About ten embryos at each stage were analysed to make sure the expression pattern was reproducible.
Data exclusions	When only a transgene insertion showed ectopic expression not present in at least three other lines, that line was disregarded and the ectopic expression was considered to be due to enhancer trap effects.
Replication	Most experiments were repeated using different antibodies and markers. For all transgenic lines at least three independent insertions were studied that confirmed reproducible expression.
Randomization	Irrelevant
Blinding	Not necessary. The mutant reporter lines were stained simultaneously with the wild type reporter, allowing the direct comparison of expression differences.

Reporting for specific materials, systems and methods

We require information from authors about some types of materials, experimental systems and methods used in many studies. Here, indicate whether each material, system or method listed is relevant to your study. If you are not sure if a list item applies to your research, read the appropriate section before selecting a response.

Materials & experimental systems

n/a	Involved in the study	
☐	☒	Antibodies
☒	☐	Eukaryotic cell lines
☒	☐	Palaeontology
☐	☒	Animals and other organisms
☒	☐	Human research participants
☒	☐	Clinical data

Methods

n/a	Involved in the study	
☒	☐	ChIP-seq
☒	☐	Flow cytometry
☒	☐	MRI-based neuroimaging

Antibodies

Antibodies used	chicken anti-GFP (Abcam 13970), rat anti-RFP (Chromotek 5F8), rabbit or mouse anti-β-Gal (Promega), rabbit anti-Dfd and anti-Labial (T. Kaufman), mouse anti-Ubx/abd-A (FP6.87), rabbit anti-HA (Abcam 9110). The following Invitrogen secondary antibodies were used: anti-chicken A488, anti-mouse A488, anti-mouse A555, anti-mouse A647, anti-rabbit A488, anti-rabbit A555, anti-rabbit A647 and anti-rat A555.
Validation	All antibodies used have been validated in innumerable publications

Animals and other organisms

Policy information about studies involving animals; ARRIVE guidelines recommended for reporting animal research

Laboratory animals	Drosophila melanogaster stocks: Bloomington Drosophila Stock Center were used: Dfd16; Scr4; lab14; P{UAS-Dfd.B}W4; P{UAS-lab.M}X2 ;P{UAS-LacZ}; Df(1)os1A; ScrC1 AntpNS+RC3 UbxMX12 34, ScrC1 AntpNS+RC3 Df109, hthP2, CyOwgen11.<br>The following lines from our laboratory were used: UAS-Ubx; UAS-AbdBm; the reporter vvl1+2 mCherry; the enhancer trap line sal-GAL4 459.2 and arm-Gal4.
Wild animals	Not used
Field-collected samples	Not used
Ethics oversight	No ethical approval is necessary for Drosophila work. Transgenic lines were manipulated in rooms prepared to contain them. We have permission from the Spanish Ministry of Agriculture to work with transgenic flies.

Note that full information on the approval of the study protocol must also be provided in the manuscript.
